# Beyond tobacco: understanding the growing burden of lung adenocarcinoma in women

**DOI:** 10.3389/or.2026.1813124

**Published:** 2026-09-02

**Authors:** Michael Christian A. Virata, Rizal Michael Ramos Abello, Edroico Mari Beltran Brillante, Jasmine Soco Interior, Patrick Reyes Relacion, Ryan Gabriel C. Molen, Eryll Paolo M. Alea, John Cedric P. Mojica

**Affiliations:** 1 Department of Pathology, Molecular and Cell-Based Medicine, Icahn School of Medicine at Mount Sinai, New York, NY, United States; 2 Department of Family Medicine, Guthrie – Robert Packer Hospital, Sayre, PA, United States; 3 College of Medicine, San Beda University, Manila, Philippines; 4 Department of Biology, College of Science, De La Salle University, Manila, Philippines; 5 Mapua School of Medicine, Mapua University Makati, Makati, Philippines; 6 College of Allied Health Professions, University of the East Ramon Magsaysay Memorial Medical Center, Inc., Quezon City, Philippines; 7 College of Public Health, University of the Philippines - Manila, Manila, Philippines; 8 School of Medicine and Public Health, Ateneo de Manila University, Pasig, Philippines; 9 Faculty of Medicine and Surgery, University of Santo Tomas, Manila, Philippines; 10 College of Medicine, University of the East Ramon Magsaysay Memorial Medical Center, Quezon City, Philippines

**Keywords:** EGFR, estrogen, estrogen receptor beta, KRAS, lung adenocarcinoma, smoking

## Abstract

Primary lung adenocarcinoma is the most common histological subtype of lung cancer. It disproportionately affects female sex, especially never-smokers. The rising incidence among females without a smoking history suggests roles for hormonal, genetic, and environmental factors. We conducted a comprehensive literature review using several databases. We examined global epidemiology data, molecular mechanisms, hormone-related pathways, and new diagnostic and therapeutic approaches. The findings show that estrogen signaling, especially via estrogen receptor beta (ERβ), contributes to oncogenesis. The mechanism involves cross-talk with *EGFR* and downstream MAPK/PI3K pathways. *EGFR* mutations appear more frequently in females. The association between KRAS and other cell cycle drivers is mixed across studies. Non-tobacco exposures, including air pollution and secondhand smoke, further increase risk among females. Diagnostic disparities persist because current screening guidelines focus on smoking. This approach excludes many high-risk never-smoking females. This review aims to narrate and synthesize growing evidence highlighting how female sex is associated with a distinct epidemiological footprint, unique molecular and biological profiles, and tailored therapeutic strategies in lung adenocarcinoma, driven by mechanisms beyond traditional tobacco exposure. Incorporating sex-specific risk assessment, hormonal level modification, and targeted gene therapies into clinical practice may improve screening and outcomes for females with lung adenocarcinoma.

## 1 Introduction

Primary lung cancer is the leading cause of cancer mortality worldwide, causing 1.8 million deaths in 2020 (1). Colorectal and liver cancers follow. Adenocarcinoma is the most common histologic subtype. In most countries, adenocarcinoma has surpassed squamous cell carcinoma as the most common NSCLC histologic subtype in many regions, in both male and females (2). Recent studies show a rising incidence of lung adenocarcinoma in perimenopausal females, including smokers. Yet, many cases occur in females with little or no tobacco exposure (3–5). These trends help explain why the classic lung adenocarcinoma profile is a middle-aged, non-smoker woman (6).

Several factors underlie this epidemiologic distribution. Building on the epidemiologic trends, at the molecular level, lung adenocarcinoma is caused by oncogenic mutations in the epidermal growth factor receptor (*EGFR*) and Kirsten rat sarcoma viral oncogene homolog (*KRAS*) genes. These gene mutations appear more often in females than in males across populations (7, 8). Estrogen may also affect the lung parenchyma. Some studies suggest that the aromatase enzyme and estrogen receptor B (ERβ) are expressed in non-small cell lung cancer (NSCLC) (9, 10).

In 2021, the USPSTF recommended annual lung cancer screening with low-dose CT. This applies to adults aged 50–80 years with a 20-pack-year smoking history. The recommendation targets current smokers or those who quit within the past 15 years (11). The guidelines refer to the largest randomized controlled trials (RCTs) to date: the NLST and NELSON trials. These focused on participants with significant smoking histories (12, 13). However, they do not address the rising incidence of lung adenocarcinoma in females who have never smoked. For example, Siegel et al. found that, out of 129,309 females with lung cancer from 2011 to 2016, 3,339 cases (11.8%) involved those with no tobacco history (5). This highlights the need for strategies to reduce missed cancers in non-smoking females, while maintaining the mortality benefit seen in smoking-based trials.

In this review, we examine the available literature characterizing the distinct clinical and biological entity of lung adenocarcinoma in females. We cover its unique epidemiology, sex-specific molecular profiles, and the role of estrogen signaling. Clarifying these factors may help explain sex differences in incidence. This may also guide diagnostic and therapeutic strategies, including anti-estrogen and *EGFR*-targeted therapy. These factors can support recommendations to improve screening policy.

## 2 Epidemiology and burden

The epidemiology of lung adenocarcinoma reflects interactions between predisposing factors such as sex, smoking status, and environment. Globally, 50.75% of lung cancer cases are adenocarcinoma, totaling to 1,259,182 cases. East Asia had the highest age-standardized incidence rate (27.12 per 100,000 people) in 2022 (14). The mean diagnosis age is 71 years. Over two decades, adenocarcinoma has replaced squamous cell carcinoma as the most prevalent non-small cell cancer (15). Despite decreased incidence and death, lung adenocarcinoma remains the leading cause of cancer mortality worldwide (16). This underlines the importance of exploring its risk factors.

Tobacco use remains the main risk factor for lung adenocarcinoma. Recent evidence links female sex to a higher incidence compared to men, possibly due to hormonal, genetic, and exposure differences. Females are diagnosed younger and more often are never-smokers, providing insight into unique factors affecting female lung cancer patients (17). The association between sex and lung adenocarcinoma incidence differs by smoking status. In Morocco, never-smoking females comprise 77.92% of adenocarcinoma cases (18), differing from patterns seen in smokers. A United States study found a significant proportion of never-smoking females diagnosed with adenocarcinoma (prevalence ratio = 1.63) (5). This evidence clarifies sex-related roles and highlights unexplored factors in diagnosis and risk analysis.

Trends in screening, histological classification, and birth cohorts reveal sex differences in lung adenocarcinoma cases. Lung cancer screening has improved, and more diagnoses occur in female never-smokers. However, eligibility for low-dose computed tomography depends only on smoking history and age, not sex (19,20). Thus, many female never-smokers remain ineligible for screening under current frameworks despite their disease burden. However, expanding clinical screening parameters requires substantial foundational data. To mitigate the clinical dangers of over-screening, including high false-positive rates and unnecessary procedures, future research must focus on developing multi-variable risk models and conducting prospective screening trials specifically targeting never-smoking populations before clinical guideline modifications can be safely implemented. Characterizing female sex through its distinct clinical and environmental presentation adds complexity to understanding sex disparities in lung cancer, especially when evaluating how biological variables intersect with smoking behaviors, case ascertainment, and environmental exposures (21). Factors like region, secondhand smoke, air pollution, and biomass exposure affect all, though air pollution shows stronger links to adenocarcinoma in female non-smokers (22).

## 3 Sex differences and risk factors

**FIGURE 1:**
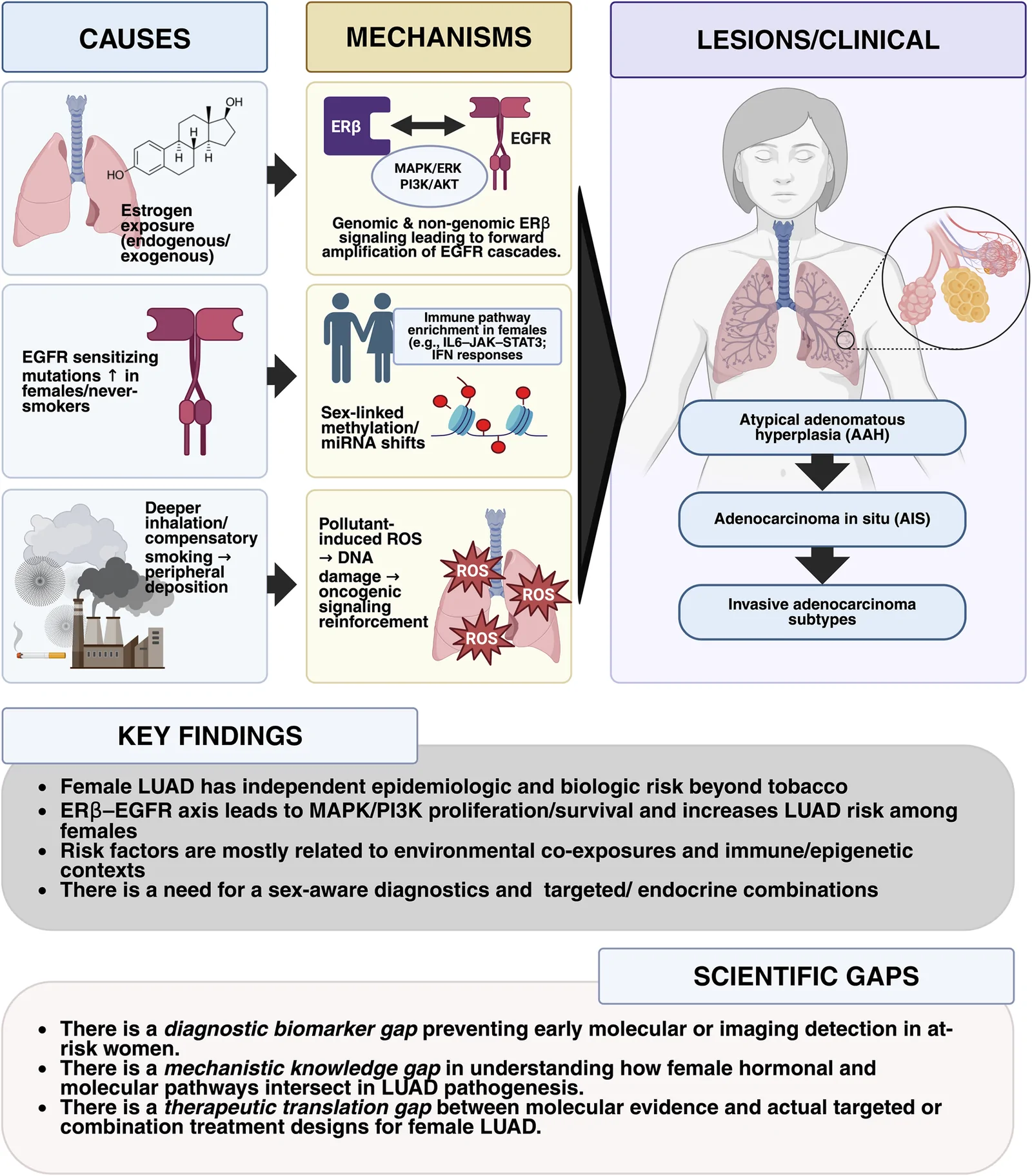
Sex-specific causes, mechanisms, and clinical progression of female lung adenocarcinoma (LUAD). Created in BioRender. Relacion, P. (2025) https://BioRender.com/p1ldbpi.

The role of estrogen has been known in different cancers, most commonly in the breast. However, newer studies suggest that the hormone may also play a role in lung adenocarcinoma. Both endogenous and exogenous estrogen exposure are linked to poorer outcomes, such as increased mortality rate. However, effects on incidence remain mixed and causal inferences are unresolved (23–25). Both normal lung parenchyma and lung tumors express ERα/Erβ as well as the G-protein-coupled estrogen receptor (See Figure 1). Their activation triggers common cancer mechanisms, including increased proliferation, apoptotic suppression, and *VEGF*-dependent angiogenesis (26). The pathway works through estrogen, affecting the genomic (nuclear ER-mediated transcription) and non-genomic pathways that activate cAMP, *MAPK/ERK*, and *AKT*. Estrogen also cross-talks with *EGFR*, leading to upregulation of cell cycle proteins such as c*-MYC, cyclin D*, and *Id* proteins, which accelerate cellular growth. In addition, the aromatase enzyme, which converts androgen to estrogen, shows elevated expression in lung tumors and non-small cell lung cancer metastasis. This correlates with higher tumor estrogen and Erβ levels (25).

The differential risk of lung adenocarcinoma between premenopausal and postmenopausal females is thought to relate to variations in circulating estrogen levels and estrogen receptor activity. Premenopausal females have higher endogenous estrogen concentrations, which can activate more estrogen receptors in lung tissue and tumors. While receptor activation has been hypothesized to promote tumor development and aggressiveness, its clinical impact remains a subject of ongoing debate. Some clinical data suggest an association between high endogenous estrogen levels and a trend toward advanced stage at presentation or a more aggressive course in premenopausal women; however, literature findings are mixed, and a definitive causal link between circulating estrogen levels and distinct postmenopausal versus premenopausal prognoses has not been firmly established. A study by Maitra et al. (2021) revealed that premenopausal females, ages 15 to 50, synthesize many estrogens and thus are at a greater risk of developing NSCLCs (27).

Normally, one would expect that lower circulating estrogen levels in postmenopausal females would reduce the stimulatory effect on lung adenocarcinoma cells. This could result in relatively slower tumor progression and potentially better clinical outcomes. However, a study by Cheng et al. (2023) reported contrasting outcomes. They found that postmenopausal females have a higher risk of lung cancer death with a longer time since menopause (23). Their study showed death risk increases by 2% for each year since menopause and by 52% for more than 20 years compared with less than 5 years since menopause. The mechanism is unclear, but it suggests that the decline in endogenous estrogen levels following menopause may influence lung cancer progression. This could be due to the loss of estrogen’s protective effects on lung tissue.

Although molecular and hormonal influences contribute to sex differences in lung adenocarcinoma incidence, behavioral and environmental exposures remain central to explaining the higher burden of this subtype among females (28, 29). In recent decades, changes in smoking behavior in females have reshaped the epidemiologic landscape of lung cancer in their population (30, 31). A key factor lies in cigarette consumption patterns. Females typically smoke fewer cigarettes than men and often use filtered or “light” varieties. These products were historically marketed as safer alternatives (32). However, strong evidence shows that such products do not reduce harm. There is no difference in lung cancer risk between people who smoke light or ultralight cigarettes and those who smoke regular ones (33–36). The design of “light” cigarettes often encourages compensatory behaviors. These include deeper inhalation, longer puff duration, and greater smoke intake. Such habits lead to increased deposition of carcinogens in the peripheral lung, where adenocarcinomas often develop (30).

Epidemiological studies consistently show that several smoking behaviors—such as duration, intensity, inhalation depth, and cumulative exposure—raise the risk of lung adenocarcinoma. These behaviors often show a clear dose–response relationship with risk. One of the earliest studies on this topic, a case–control study from Spain, found that deeper inhalation increased lung cancer risk, even after accounting for cigarette quantity and duration (37).

More recent population-based evidence reinforces this finding. A large French case–control study by Rusmaully et al. (2021) found females with the longest duration and highest intensity of smoking were over twelve times more likely to develop lung cancer than those with minimal exposure (OR = 12.64; 95% CI: 8.50–18.80) (38). Notably, risk was similar across filter types, indicating the presence of compensatory behaviors, as previously discussed.

Another important risk factor for lung adenocarcinoma in females is secondhand smoke exposure, especially for non-smoking females. Household exposure to smoking family members was once a major source of involuntary carcinogen exposure. This was particularly common before the adoption of indoor smoking restrictions. Systematic reviews and meta-analyses show that secondhand smoke increases lung cancer risk. Risk rises alongside the duration and intensity of exposure (39–41).

Beyond direct and passive tobacco exposure, environmental and occupational risks also contribute. In some regions, lung adenocarcinoma rates are rising among never-smoking females. Here, exposures such as indoor air pollution from cooking fumes, biomass fuels, and specific workplace hazards like silica, welding fumes, and radon play a role (42–44). Occupational exposure to asbestos, arsenic, and other carcinogens, once mainly linked to male-dominated industries, now increasingly affects females as labor markets change (45–47).

## 4 Molecular and hormonal mechanisms

Environmental factors also play a major role in the pathogenesis of lung adenocarcinoma. There may also be sex-specific factors, including genetic ones, involved in the development of this condition. The most common mutations found in patients with lung adenocarcinoma (regardless of sex) include alterations in *EGFR, KRAS, ALK*, and *MET*, listed in descending order of frequency (48). Multiple studies have examined the frequency of these mutations and compared their prevalence between males and females (see Table 1 for a summary).

**TABLE 1:** Oncogenic mutations involved in the pathogenesis of lung adenocarcinoma and their relative occurrence between sexes.

Oncogenic mutations			
Gene	Normal function	Type of mutation	Sex difference
Epidermal growth factor receptor (EGFR)	• Initiates intracellular pathways that lead to cell proliferation, cell differentiation, angiogenesis, metastasis and anti-apoptosis	• Exon 19 deletions • Exon 21 point mutation	• (20): Females > Males • (48): Females > Males but not significant • (49): Females > Males (significant)
KRAS	• Regulates cellular signalling cascades (including RAF-MEK-ERK pathway) that subsequently regulates cell proliferation, survival and differentiation	• Transition mutation (i.e. purine to purine; pyrimidine to pyrimidine) in codons 12 and 13	• (48): Males > females (significant) • (52): Males > females (significant) • (7): Females > Males (significant) • (60): Higher in non-smoker males, higher in smoker females (not significant) • (49): Males > females
ALK	• Autophosphorylation and activation of signaling pathways (PI3K-AKT, mTOR, MAPK, and STAT3) consequently leading cell proliferation, survival and differentiation	• EML4-ALK Translocation • gene fusions	• (56) and (48): Males > females (Not significant) • (82): No relation to sex
MET	​	• Exon 14 splice site mutations • DNA amplification • MET copy number gain	• (58) females > Males
BRAF	• Activates the RAS-RAF-MEK-ERK-MAP signal pathway that mainly functions to regulate cell proliferation, differentiation, and survival	V600E mutations	• (48): Males > females (Not significant) • (48): No significant difference between sexes

Yellow - more common in females; Blue - more common in males; Green - similar incidence or insignificant difference.

### 4.1 Most common oncogenic mutations in lung adenocarcinoma

#### 4.1.1 Epidermal growth factor receptor (*EGFR*) mutations

Across several published articles, *EGFR* mutation has been consistently implicated in the pathogenesis of lung adenocarcinoma. It is highly expressed in lung cancer cells (Gaur et al., 2018). Alterations in this gene have been reported to occur more frequently in non-smoking females than in their male counterparts (48, 49). *EGFR* is a receptor with a tyrosine kinase domain. When activated, it initiates cell proliferation and differentiation. In addition to these intracellular effects, activation of the *EGFR* also stimulates angiogenesis, metastasis, and anti-apoptosis (50). The most common mutations (involving exons 19 and 21) are considered sensitizing mutations of *EGFR* (8). These mutations lead to the perpetual activation of *EGFR* (51). This further augments the effects of the *EGFR* tyrosine kinase, consequently leading to tumors, including lung adenocarcinoma.

#### 4.1.2 *KRAS* mutations

The *KRAS* gene, when activated, initiates a downstream signaling cascade. This includes the *RAF-MEK-ERK* pathway, which regulates cell proliferation, survival, and differentiation. A mutation in this gene leads to uncontrolled cell proliferation and, eventually, tumor formation. According to Judd et al. (2021), the most common mutation in the *KRAS* gene among non-smokers is a transition mutation at codons 12 and 13. In these mutations, a purine is replaced by a purine, or a pyrimidine is replaced by a pyrimidine (7).

Whether *KRAS* mutations occur more frequently in females remains unresolved, as studies have reported differing results, specifically, discovering a higher frequency in males (with significant differences compared with females (48, 49, 52). However, Judd et al. (2021) reported the opposite, finding a significantly higher frequency in females (7). These conflicting results highlight ongoing uncertainty in the literature.

#### 4.1.3 Anaplastic lymphoma kinase (*ALK*) mutations

When physiological ligands bind to *ALK*, the receptor dimerizes and undergoes self-phosphorylation. This activation turns on multiple intracellular signaling cascades, including the *PI3K-AKT, mTOR, MAPK, and STAT3* pathways (53). This results in cellular proliferation and differentiation. Studies discovering the association between the *ALK* gene and sex have been very inconsistent. In lung adenocarcinoma, three main types of *ALK* gene alterations have been found: rearrangements, point mutations, and gene amplifications, with the most common being *ALK* gene translocation (54). The implication of female sex in *ALK* gene mutation is not well understood, and the results of different studies are conflicting. A meta-analysis of the *EML4-ALK* fusion gene found that females had significantly higher fusion gene positivity (55). In contrast, another study identified around 350 cases of non-small cell lung carcinoma with *ALK* rearrangement and *ALK* germline status. It found no significant difference (p = 0.16) between male and female sex (56).

#### 4.1.4 *MET* mutations

The MET tyrosine kinase pathway is often upregulated in lung adenocarcinoma and other cancers. It normally regulates cell growth, movement, and survival. When dysregulated, it drives tumor proliferation, invasion, metastasis, and angiogenesis (57). A study reported that MET 14 skipping was more common in females, non-smokers, older individuals, and those with lung adenocarcinoma, compared with other demographic groups (58). While this could offer a new therapeutic window for those with MET-mutant NSCLC, the same study found that the rate of MET mutations is very low (1.9%), compared with *EGFR* mutations, which account for 74.5% of never-smoking lung adenocarcinomas.

#### 4.1.5 *BRAF* mutations

*BRAF*, or the B-Raf gene (V-Raf Murine Sarcoma Viral Oncogene Homolog B), is a Serine/Threonine kinase. Stimulation of the Ras-GTP complex leads to the phosphorylation and activation of *MEK* and *ERK*. Activation of this pathway regulates cell proliferation, differentiation, and survival. *BRAF* gene mutations, most commonly the V600E mutation, lead to continuous activation of the *RAS-RAF-MEK-ERK-MAP* signaling pathway (59). This subsequently leads to augmented cell proliferation, infiltration, and metastasis.

Intuitively, these mutations are expected to be more common in females than in males. However, more recent studies, as previously discussed, reveal a different pattern. In the case of *KRAS*, studies showed mixed results. This suggests there are other genetic factors that underlie the propensity of lung adenocarcinomas to develop in female non-smokers. Li et al. (2023) explored this possibility and found that, in females, the affected genes are mostly related to the immune pathway (49).

### 4.2 Mutations with differential effects

Other genes worth noting are those that are expressed differently between the two sexes. This differential expression leads to two distinct effects depending on the patient’s sex, as studied by Xu et al. (2022). These include the *KRT16, ERBB4*, and *NTF4* genes, as well as several others involved in the *MAPK/PI3K* signaling pathway. The *KRT16* gene, a component of the estrogen receptor signaling pathway, was noted to increase the hazard ratio in females to twice that of males expressing the same gene (60). On the other hand, decreased expression of *ERBB4*, a member of the *EGFR* family, is associated with a poor prognosis in males but may be associated with prolonged survival in females. Lower expression of *NTF4,* or Neurotropin-4 (a member of the neurotrophic factor family), shows the opposite effect. It brings a worse prognosis when found in females.

## 5 Screening and diagnostic approach

### 5.1 Biomarkers

In general, biomarkers are essential for assessing prognosis and response to targeted treatments in patients with cancer (61). Identifying biomarkers is also essential for selecting the most effective treatment, especially given molecular profile differences between sexes in lung adenocarcinoma. According to Li et al. 2023, mutations biased toward females (excluding *EGFR*) were mostly identified on the X chromosome. In contrast, mutations biased toward males were located on autosomal chromosomes. Furthermore, a higher prevalence of *EGFR* mutations was observed in nonsmoking females compared with male nonsmokers, consistent with previous findings (62, 63). Females showed significant enrichment in immune-related pathways, including IL6-JAK-STAT3 signaling, responses to interferon alpha and gamma, and the inflammatory response (49). Mosleh et al. 2025 also found that females had markedly elevated KRAS signaling and HER-mutant lung cancers. This might be explained by higher cytochrome *CYP1A1* gene expression in females’ lungs (64). This gene enhances the activation of carcinogens. There is also overexpression of the estrogen receptor, especially ERβ, which is the most common variant. ERβ is especially high in premenopausal females, according to numerous studies (65, 66).

The *ALK* gene, included in comprehensive biomarker testing for patients suspected of having lung adenocarcinoma, encodes the tyrosine kinase receptor *ALK*. According to Sánchez-Ares et al. 2016, *ALK* rearrangements are found in 1.9%–6.8% of patients with non-small cell lung cancer (NSCLC) (67). Its detection may be a significant diagnostic tool for patients with suspected cases. Most mutation carriers are young people, females, and non-smokers. There are currently three methods to detect *EGFR* and *ALK* gene rearrangements: (1) real-time PCR (RT-PCR), (2) immunohistochemistry (IHC), and (3) fluorescence *in situ* hybridization (FISH). FISH is the gold standard for detecting *ALK* gene rearrangement in NSCLC. The prospective use of the ddPCR-based plasma genotyping tool to guide clinical decisions is supported by its capacity to detect *EGFR* mutations swiftly and accurately in a clinical setting, as reported by Sacher et al. 2015 (68). However, according to the latest guidelines, IHC stains using FDA-approved antibodies are a suitable alternative due to their ease of access and cost-effectiveness (69).

Recently, the United States FDA authorized an IHC assay that uses the *ALK* D5F3 antibody as a companion diagnostic for patients with *ALK*-rearranged NSCLC. Significantly, the *ALK* D5F3 antibody IHC appears to be both a competitor and the most essential assay for *ALK* testing. Brainard et al. (2019) reported that ultrasensitive *ALK* IHC using the rabbit monoclonal antibody D5F3 can detect the unique *ALK* protein generated by this gene rearrangement (70). As a result, patients with *ALK*-positive NSCLC identified by immunostaining with clone D5F3 qualify for *ALK* inhibitor therapy (71).

### 5.2 Early screening

Different guidelines suggest different starting ages for lung cancer screening, but the most frequent range is from 50 to 55 years old, with females generally diagnosed sooner. In 2021, the USPSTF recommends annual screening for lung cancer with a low-dose computed tomography (LDCT) scan in adults aged 50–80 years who have a 20-pack-year smoking history and currently smoke or have quit within the past 15 years (11). Building on these recommendations, a study by Wang et al. (2025) reported that LDCT of peripheral lung areas may be more advantageous for females, as it can detect peripheral nodules or adenocarcinoma lesions with indistinct edges (72). Furthermore, evidence from the National Lung Cancer Screening Trial (NLST) in the United States has shown that annual LDCT screening for 3 years lowered the risk of lung cancer mortality in female patients at risk and heavy smokers, both current and former, and proved more effective than chest x-rays in identifying early-stage lung cancer. Finally, insufficient awareness might contribute to the low rates of lung cancer screening among females, many of whom do not know that lung cancer is the most frequently diagnosed cancer in females (73).

## 6 Therapeutic implications

### 6.1 Endocrine therapies

Estrogens are steroid hormones involved in processes such as the development of the female reproductive system, the regulation of metabolism, and the formation of secondary sexual characteristics. Beyond these functions, estrogen also affects lung development and function through its widely expressed receptor in lung epithelium (74). This finding emphasizes the potential for endocrine therapy in lung adenocarcinoma, particularly targeting estrogen receptor β (ERβ) and its signaling pathways. Importantly, unlike breast cancer, where ERα predominates, lung tissues mainly use ERβ, which governs cell proliferation and angiogenesis (See Table 2). Furthermore, subcellular location matters: nuclear ERβ associates with better survival, while cytoplasmic ERβ associates with aggressive behavior (75). Notably, intratumoral aromatase produces estrogen locally, sustaining ERβ signaling even after menopause and driving tumor growth in female patients (76, 77). Collectively, these findings provide a strong rationale to explore endocrine therapy for lung adenocarcinoma, especially in females.

**TABLE 2:** Endocrine-targeted therapeutic strategies in female lung adenocarcinoma.

Therapy class	Mechanism/Target	Key pathways affected
Aromatase inhibitors	Block intratumoral estrogen biosynthesis (aromatase inhibition)	ERβ, MAPK, *AKT*, *EGFR*, *CXCR4*
Selective Estrogen receptor Modulators	Modulate ER activity (agonist/antagonist effects, mainly ERβ)	ERβ, MAPK, *AKT*
Selective Estrogen receptor Degraders	Promote ER degradation, abolishing transcriptional activity	ERβ, MAPK/AKT transcriptional programs
Combination: Endocrine + *EGFR* TKIs	Dual blockade of ERβ and *EGFR* pathways to disrupt feed-forward oncogenic loop	ERβ–*EGFR* crosstalk, MAPK, PI3K
Combination: Endocrine + ICIs	Reduce ERβ-driven PD-L1 expression, enhancing anti-tumor immunity	ERβ–PD-L1 axis, immune checkpoints

#### 6.1.1 Aromatase inhibitors

Aromatase inhibitors suppress estrogen biosynthesis in two mechanisms: first, via irreversible steroidal inhibitors such as exenastane, which form a strong bond with the aromatase; and second, via reversible competitive inhibition with non-steroidal inhibitors such as anastrazole, which reduce estrogen synthesis by inhibiting the aromatase activity domain. In preclinical models for lung adenocarcinoma, these drugs reduce proliferation and tumor growth, and promote apoptosis, especially in tumors with high aromatase levels or ERβ expression, both *in vivo* and *in vitro* (78). A phase Ib trial of exemestane combined with chemotherapy (NCT01664754) showed acceptable tolerability. However, a phase II study of letrozole combined with everolimus was complicated by severe pulmonary toxicity.

#### 6.1.2 Estrogen metabolism modulation

In addition to inhibiting estrogen synthesis, estrogen metabolism can be manipulated to reduce hormone bioavailability. Estrogen sulfotransferase (SULT1E1) catalyzes the sulfonation of estrogens, thereby decreasing the levels of active hormones. Studies have shown that dexamethasone, a synthetic glucocorticoid, induces SULT1E1 expression. This decreases estrogen activity and suppresses the proliferation of lung adenocarcinoma (79).

#### 6.1.3 Selective Estrogen receptor degraders (SERDs)

SERDs, such as fulvestrant, act by destabilizing the estrogen receptor beta (ERβ) and blocking its transcriptional activity. In NSCLC models, fulvestrant has been found to slow tumor growth, reverse epithelial–mesenchymal transition, and trigger apoptosis, especially in tumors with high ERβ levels. SERDs also enhance the effects of *EGFR* inhibitors in xenograft models by further suppressing cell growth, increasing cell death, and causing more significant tumor regression than either drug alone (80). These observations indicate that SERDs may be valuable for combined approaches targeting both hormone and kinase pathways. A study by Hamilton et al. demonstrated that fulvestrant reduces features characteristic of mesenchymal cells and increases tumor sensitivity to chemotherapy and immune-mediated attack in lung cancer cells (81).

#### 6.1.4 Selective Estrogen receptor modulators (SERMs)

Estrogen, acting via the *CXCR4/CXCL12* signaling pathway, has been shown to stimulate *CXCR4* overexpression, thereby increasing tumor cell migration. SERMs like tamoxifen bind to ERβ, altering its activity as a partial agonist or antagonist, depending on the tissue. In lung adenocarcinoma models, these drugs lower *CXCR4* expression, thereby reducing cell movement and reversing cisplatin resistance by inhibiting *PKC/c-FOS* signaling (83).

### 6.2 *EGFR* targeting pathway

Oncogenic drivers commonly associated with lung adenocarcinoma in the female population intersect with estrogen signaling in *EGFR*-mutated tumors (84, 85). *EGFR* tyrosine kinase inhibitors (TKIs) are the cornerstone of treatment for tumors with sensitizing *EGFR* mutations. ERβ activation particularly enhances *MAPK* and *PI3K/AKT* pathways, creating a feed-forward loop that amplifies *EGFR* signaling. Gefitinib was among the first agents tested for *EGFR*-expressing NSCLC, targeting the ATP cleft within *EGFR*, which is overexpressed in 40%–80% of NSCLC cases. However, it was later found out that only tumors with somatic mutations in the tyrosine kinase domain of the *EGFR* gene responded to this. Hence, driver mutation testing for newly diagnosed, advanced NSCLC cases has become the standard of care. Consequently, the acquisition of the T790M mutation at the *EGFR* ATP-binding site hinders the binding of first-generation *EGFR* TKIs, leading to recurrence within a year. Hence, the development of second-generation irreversible pan-*EGFR* inhibitors, afatinib and dacomitinib, has been proven to be effective in suppressing the T790M mutation (85). At present, osimertinib is recognized as the first-line standard of care for patients with advanced *EGFR*-mutated NSCLC with confirmed T790M mutation-positive, due to its greater potency and selectivity against both typical *EGFR* activating mutations and the T790M resistance mutation (86, 87). Erlotinib, gefitinib, and afatinib are also approved as first-line treatments for targetable *EGFR* alterations, with median progression-free survival (PFS) of 9.2–13.1 months (85, 88, 89). The subgroup with an *EGFR* exon 19 deletion has better PFS with dacomitinib than with erlotinib.

CNS metastasis is a common site of disease progression in *EGFR*-positive NSCLC, leading to failure of the first-line TKIs. AZD3759 is a potent CNS-penetrant *EGFR* TKI currently being evaluated, along with osimertinib, in the BLOOM phase I study among patients with progressive CNS metastasis who have already received *EGFR* TKI therapy. It exhibited activity in 20 patients with measurable brain metastases evaluable for RECIST assessment, among whom 8 had tumor shrinkage in the brain, 3 had confirmed partial response, and 3 had unconfirmed partial response. In 12 patients taking osimertinib, 7 had radiological partial response, 2 had stable disease, and 3 were not evaluable after 12 weeks (84, 90).

### 6.3 *ALK* and *ROS* pathway targeting

*ALK* mutations are found in 1.9%–6.8% of patients with non-small cell lung cancer (NSCLC) (67). Crizotinib, approved by the FDA in 2011 for advanced NSCLC with *ALK* rearrangements, showed notable efficacy in clinical trials despite being initially developed as a MET inhibitor. However, to overcome resistance to crizotinib and enhance CNS efficacy, second-generation TKIs have been developed, such as alectinib and brigatinib. Among *ALK*-positive patients, alectinib showed a significant improvement in disease-free survival compared with platinum-based chemotherapy in the ALINA trials (91). Lorlatinib, a highly-effective third-generation, and selective *ALK/ROS1 TKI* was granted accelerated FDA approval in 2018 for NSCLC patients with *ALK* rearrangements who have overcame on first-line alectinib, brigatinib, or crizotinib therapy, wherein on CROWN trial phase III showed a 72% reduction in the risk of disease progression or death, with a markedly prolonged time to CNS progression (no CNS progression in 12 months, 96% vs. 60%) and more recently a 5-year PFS of 60% vs. 8% (92, 93).

Subsequently, pharmacologic agents for *ALK*-positive metastatic NSCLC have also been recommended for use in the treatment of *ROS1*-positive metastatic diseases, as, despite being an independent receptor tyrosine kinase, *ROS1* shares approximately 70% homology with the kinase domain of ALK, as evident by the ALK inhibitor crizotinib (94, 95). This achieved an objective response rate of 72%, with a median OS of 51.4 months. An oral tyrosine kinase inhibitor, Entrectinib, demonstrated efficacy as first-line therapy in treating ROS1-positive metastatic NSCLC patients in phases I and II of the STARTRK-2, STARTRK-1, and ALKA-372-001 trials, surpassing crizotinib but at the cost of a higher incidence of adverse effects. For *ROS1*-positive patients with metastatic NSCLC, the priority first-line treatments include entrectinib, tasetrectinib, or repotrectinib, with crizotinib, ceritinib, and lorlatinib as alternative options; for those with brain metastases, entrectinib or repotrectinib may be appropriate (96).

### 6.4 *KRAS* mutation targeting

*KRAS* is one of the most commonly mutated oncogenes, accounting for approximately 30% of lung adenocarcinoma (97). Although the targeted immunotherapy landscape for patients with NSCLC and KRAS mutations remains unclear, PD-L1 levels may guide selection of the appropriate pharmacologic approach. *KRAS* inhbitors such as sotorasib or adagrasib are treatment options among such patients are approved treatments in patients with NSCLC. However, post marketing safety are limited (98). In addition, sequential use of anti-PD-(L)1 and sotorasib therapy is associated with increased risks of hepatic toxicities and occurrence of non-hepatic severe adverse events, which necessitates a 30-day gap between treatments (99). However, early evidence has shown that the combination of adagrasib and pembrolizumab is a safe and effective regimen for newly diagnosed NSCLC with a *KRAS* mutation (100).

### 6.5 *BRAF* mutation targeting

*BRAF* is a mediator of the KRAS downstream signaling pathway, which activates the MAP kinase pathway. *BRAF* mutations are found in 1%–2% of NSCLC cases, are associated with smoking, and are among the resistance mechanisms associated with *EGFR* TKIs (101). Currently, vemurafenib and dabrafenib are approved for *BRAF* V600E-positive malignant melanomas, although single-agent activity in *BRAF* V600E-positive NSCLC is limited. Among patients with metastasis, dabrafenib combined with trametinib or encorafenib + binimetinib may be preferred based on phase II trials (101). In addition, retrospective studies suggest that patients with advanced NSCLC with *BRAF* mutations might also benefit from PD-1/PD-L1 inhibitor therapies, likewise, immunotherapy regimens based on Immune checkpoints inhibitors in combination with chemotherapy can be considered as treatment among patients with a low disease burden or high PD-L1 levels, and can also represent potential treatment options among patients whose condition progresses after therapy with *BRAF* inhibitors. Additionally, sequential inhibition of *BRAF* and downstream MEK is an active area of lung cancer research following encouraging results in melanoma patients (84).

## 7 Future directions

While the current lung cancer screening strategy fails to capture the demographic shift toward never-smoking females, a direct clinical expansion of screening criteria presents a significant challenge due to the risk of over-screening. Bridging the gap between our current epidemiological knowledge and safe clinical implementation requires robust intermediary steps. Specifically, future clinical efforts should focus on robust risk-stratification modeling and dedicated screening trials for never-smokers to ensure that any potential guideline adjustments maximize mortality benefits while strictly minimizing diagnostic harms. The USPSTF’s smoking-focused criteria, based on male-dominant trials, fail to account for premenopausal and postmenopausal risk differences or potential estrogen-related cancer pathways. Furthermore, insufficient data on female-specific risk factors, such as reproductive history and occupational exposures, delay detection and care.

To build upon these recommendations, future trials and studies on lung cancer research should perform sex-stratified analyses alongside hormonal and environmental risk factors that are more relevant to females, as detailed above. Furthermore, as indoor air pollutants are increasingly affecting females, environmental health policies should be integrated into lung cancer prevention and health promotion strategies.

Shifting focus to implementation, there are barriers to the uptake of these new guidelines, promising molecular diagnostic tools, and personalized medicine. One challenge may be the explosion in methodologies and technologies to apply and interpret, and the burden this places on clinicians’ and diagnosticians’ workloads (102). Hartley-Blossom and colleagues (2024) reported that despite the updated USPSTF guidelines, a small fraction of the eligible patients undergo screening. These may be linked to individual-level, provider-level, and structural-level barriers (103).

A persistent barrier is that both providers and individuals often lack awareness of updated guidelines and the shifting epidemiology of lung cancer in never-smoking females. As a result, providers may not initiate essential screening conversations. Existing studies show that discussion and awareness rates remain low, particularly among females and non-smokers, limiting their access to appropriate screening despite their potential risk.

Clinicians should proactively initiate discussions about lung cancer screening, highlighting risks even for never-smoking females. Integrating lung cancer screening with established cancer screenings, such as breast cancer diagnostics, may further increase uptake.

This integrated approach recognizes that breast cancer is the most common non-skin cancer in females, and mammography is often used for its evaluation (104). With over 70% of eligible females screened for breast cancer (105), recommending LCS at the same time could help raise the lower rates of it. Yue et al. (2025) surveyed females knowledge, attitudes, and perceptions of LCS, finding that 58% lacked knowledge about LCS eligibility and 47% had cost concerns; still, after screening, 84% were likely to have dual screening again, and 93% found the decision-making visit helpful (106). Jani et al. (2023) also observed an increase in LCS enrollment among females scheduled for mammography following targeted outreach (107). However, self-referral for LCS was 0% in one study (108), suggesting these discussions must be initiated by providers. These findings highlight how providers can boost LCS uptake by linking it to established screenings.

## 8 Conclusion

Accumulating evidence indicates that lung adenocarcinoma in women exhibits a distinct clinical and epidemiological profile that cannot be entirely explained by traditional tobacco exposure. This association appears to be influenced by a complex interplay of hormonal signaling, genetic predisposition, and differential environmental exposures. Notably, a proposed key mechanism involves estrogen receptor β (ERβ), which interacts with the epidermal growth factor receptor (EGFR). Through this interaction, downstream signaling cascades such as the MAPK and PI3K pathways are activated, potentially contributing to abnormal cell growth and promoting tumorigenesis. These findings offer valuable insights for future diagnostic and treatment approaches. However, current screening remains heavily reliant on smoking history, meaning many at-risk female never-smokers may be overlooked under existing frameworks. As a result, developing multi-variable, sex-aware risk assessments represents a critical avenue to investigate this clinical gap. In parallel, emerging evidence supports further evaluation of endocrine therapies, such as aromatase inhibitors, SERMs, and SERDs, in combination with targeted therapies. Taken together, this growing literature highlights the need to evaluate future screening eligibility criteria, conduct more clinical trials that account for sex-specific analyses, and take policy action on indoor and ambient air quality, with the ultimate goal of improving outcomes.
